# Transcriptomic and experimental identification of immune- and telomere-related genes in pelvic organ prolapse

**DOI:** 10.3389/fphar.2026.1759552

**Published:** 2026-05-22

**Authors:** Rong Ma, Jingde Wu, Jingwei Gong, Zumei Pei, Jian Kang, Haiyan Xu, Wengang Yang, Xiande Huang, Chaoming Li

**Affiliations:** 1 Department of Urology, Gansu Provincial Hospital, Lanzhou, China; 2 Gansu University of Chinese Medicine First School of Clinical Medical, Lanzhou, China; 3 Department of Urology, Longnan First People’s Hospital, Longnan, China

**Keywords:** immune cell, key genes, pelvic organ prolapse, telomere, transcriptome sequencing analysis

## Abstract

**Background:**

Pelvic organ prolapse (POP) is a prevalent disease among women, and immune cell and telomere have potential associations with the pathogenesis of POP. The identification and validation of immune cell-related genes (ICRGs) and telomere-related genes (TRGs) in POP are of great significance for elucidating its mechanisms, screening diagnostic key genes, and identifying therapeutic targets.

**Methods:**

In this study, first, ICRGs were obtained based on immune infiltration and WGCNA; key genes related to immune cell and telomere in POP were identified from public-database transcriptome data through differential expression analysis, machine learning, expression level analysis and ROC analysis. Subsequently, a comprehensive analysis including nomogram construction, correlation analysis, GSEA, molecular regulatory network construction, and drug prediction explored the molecular mechanisms of these key genes in POP.

**Results:**

We identified 864 differentially expressed genes (DEGs), including 833 upregulated and 31 downregulated genes. The intersection of DEGs, telomere-related genes (TRGs), and immune cell-related genes (ICRGs) yielded six candidate genes. Machine learning further pinpointed CCNL1 and NAMPT as key biomarkers, which were significantly upregulated in POP samples (p < 0.05) and validated by RT-qPCR. Subsequently, a comprehensive analysis revealed their diagnostic potential via a nomogram (AUC: 0.847), a strong positive correlation (r = 0.78, p < 0.001), and enrichment in pathways such as ubiquitin-mediated proteolysis (GSEA). Molecular regulatory network construction predicted interactions with 21 key nodes and 28 interactions, and drug prediction identified 14 potential therapeutic compounds. These findings provide a theoretical basis for understanding POP pathogenesis and identifying novel therapeutic targets.

**Conclusion:**

This study identifies CCNL1 and NAMPT as novel immune- and telomere-related biomarkers for POP. These findings provide potential targets for diagnostic development and lay a computational foundation for future therapeutic strategies, although experimental validation is required to confirm their causal roles.

## 1 Introduction

Pelvic organ prolapse (POP) is defined as the descent of pelvic organs resulting from the impairment of supportive connective tissues (Collins and Lewicky-Gaupp, 2022). It is often accompanied by a sensation of vaginal heaviness, as well as urinary, defecatory, or sexual dysfunction (Zhou et al., 2023). It affects approximately 3%–6% of women, and the prevalence is rising due to the aging population (Bugge et al., 2020; van der Vaart et al., 2022). Established risk factors include pregnancy, vaginal delivery, advanced age, and obesity (Zarzecka et al., 2024; Brito et al., 2022).

Current management strategies for POP include watchful waiting, pelvic floor physical therapy, pessary use, and surgery. Both pessary placement and surgical intervention are effective treatment options for symptomatic cases, whereas asymptomatic women are generally advised to undergo clinical observation only (Raju and Linder, 2021). Although the use of pessaries and surgical interventions are effective, studies have shown that there is still a risk of complications such as pain and excessive vaginal discharge (van der Vaart et al., 2022). Therefore, exploring novel therapeutic strategies and elucidating the underlying molecular mechanisms are of significant clinical importance (Zhang et al., 2024).

Telomeres are specialized nucleoprotein structures at chromosome ends. They protect chromosome integrity and prevent improper activation of the DNA damage response mechanism (Shi et al., 2024). Abnormal telomere dynamics are linked to various health issues, such as cancer, cardiovascular disease, and poor mental health (Li S. C. et al., 2022). Telomerase is a ribonucleoprotein complex that can delay telomere shortening. Its core components include the telomerase RNA component (TERC), the catalytic subunit telomerase reverse transcriptase (hTERT), and the dyskerin protein (Zhang et al., 2023). Research indicates that telomere shortening accompanies immune cell aging, and physical exercise can upregulate telomerase activity, thereby attenuating telomere loss in immune cells (Kim et al., 2025). Studies have further revealed that telomere length varies among lymphocyte subpopulations with distinct functions and changes with age. Accelerated telomere shortening has been documented in several immune dysregulation syndromes compared to healthy controls (Katerina, 2024). Telomere damage promotes vascular smooth muscle cell senescence and facilitates immune cell recruitment following vascular injury (Uryga et al., 2021). In the context of pelvic support, telomere shortening in fibroblasts leads to cellular senescence and a reduced capacity for collagen synthesis and repair, thereby compromising the structural integrity of connective tissues (Saraswati et al., 2024). These findings demonstrate that telomere dysfunction may contribute to the pathogenesis of tissue degenerative disorders by modulating immune-inflammatory responses.

In the pathological context of POP, multiple cell types, including intrinsic pelvic floor cells and various immune cells, have been proven to play crucial roles in its pathogenesis (Liu et al., 2024). These immune cells contribute to POP pathogenesis not only through senescence but also by secreting pro-inflammatory cytokines and matrix metalloproteinases (MMPs), which disrupt the extracellular matrix (ECM) homeostasis and impair tissue tensile strength (Miao et al., 2023). Organismal aging is accompanied by the accumulation of senescent cells, which in turn leads to the decline of tissue function. Telomere shortening and damage are well-defined drivers of cellular and organismal aging (Rossiello et al., 2022), and immune cell aging has been recognized as a risk factor for POP.

Therefore, the identification and validation of telomere-related genes (TRGs) and immune cell-related genes (ICRGs) in POP are of considerable scientific and clinical importance. We hypothesized that specific genes intersecting both pathways would serve as key drivers of POP pathogenesis. Such efforts may enhance our understanding of the molecular mechanisms underlying POP, reveal novel diagnostic biomarkers and therapeutic targets, and ultimately contribute to improved treatment strategies that alleviate symptoms, enhance quality of life, and reduce the socioeconomic burden associated with POP.In this study, based on public transcriptomic data, we identified key genes associated with both telomere pathways and immune cells in POP through immune infiltration analysis, weighted gene co-expression network analysis (WGCNA), differential expression analysis, and machine learning. Further expression validation and ROC analysis confirmed their diagnostic value, and a risk prediction nomogram was developed. Additionally, Gene Set Enrichment Analysis (GSEA), molecular regulatory network construction, and drug prediction were performed to explore potential underlying mechanisms and therapeutic agents, providing a theoretical foundation for future clinical research.

## 2 Materials and methods

### 2.1 Data collection

Gene expression information of the GSE12852 and GSE53868 datasets related to POP was retrieved from the GEO. Specifically, GSE12852 (training set) (platform: GPL2986) included 8 POP samples and 9 control tissue (uterosacral) samples (Li Y. et al., 2023). GSE53868 (validation set) (platform: GPL18142) included 12 POP samples and 12 control tissue (vaginal wall) samples (Li Y. et al., 2023). Additionally, 2023 TRGs were obtained from the literature (Ling et al., 2024) (Supplementary Table 1). Although samples are limited and tissues differ (uterosacral ligament vs. vaginal wall), these are the primary GEO resources profiling POP vs. controls. We included both to maximize power, and cross-tissue validation supports the broad relevance of our findings.

### 2.2 Differential expression analysis

To identify DEGs between POP and control samples in the GSE12852 dataset, differential expression analysis was performed using the limma package (v 3.56.2) (Ritchie et al., 2015), with the threshold set as p < 0.05 and |log_2_FC| > 0.5 balanced statistical and biological significance for this small-scale exploratory study. The top 10 upregulated and downregulated genes sorted by |log_2_FC| values were visualized via a volcano plot plotted using the ggplot2 package (v 3.5.1) and an expression heatmap plotted using the pheatmap package (v 1.0.12) (Yuan et al., 2024).

### 2.3 Acquisition of immune cell-related genes

In all samples of the GSE12852 dataset, the xCell algorithm (v 1.1.0) (Aran et al., 2017) was used to evaluate the relative infiltration levels of 64 types of immune cells in POP tissue samples and control tissue samples (Liang et al., 2022), with visualization performed using the ggplot2 package (v 3.5.1). Additionally, the Wilcoxon test was applied to compare differences in immune cell infiltration between POP and control tissue samples (p < 0.05). Immune cells with significantly different infiltration abundances were screened out, designated as differential immune cells, and their visualization was conducted using the ggplot2 package (v 3.5.1).

To obtain module genes associated with the infiltration abundance of differential immune cells, WGCNA was performed on the infiltration abundance of differential immune cells in all samples of the GSE12852 dataset using the WGCNA package (v 1.73) (Langfelder and Horvath, 2008), following these steps:

First, to determine the overall correlation between POP and control tissue samples, hierarchical clustering analysis of GSE12852 was performed using the hclust function.

Next, to identify an appropriate soft threshold, the pickSoftThreshold function was used to determine the soft threshold for the clustered samples. A suitable soft threshold power was selected from the range of 1 to 30, which defined the threshold for gene-gene correlation. The relationships between the soft threshold β and the scale-free network evaluation coefficient *R* ^2^, as well as between the soft threshold power and average connectivity, were established. After setting the target threshold (*R* ^2^ = 0.80), soft thresholds exceeding the red cutoff line and those with connectivity close to 0 were screened. Generally, the first value crossing the red line was chosen as the optimal soft threshold, as the network at this point best fits the scale-free network distribution and holds biological significance.

Subsequently, to hierarchically partition data into different categories, the hierarchicalCluster function was used to perform topological overlap matrix (TOM)-based hierarchical clustering on the clustered samples, constructing a clustering dendrogram to identify modules and obtain clustered modules. Each module included a minimum of 40 genes (minModuleSize = 40), with a module merging parameter of mergeCutHeight = 0.25.

Then, to screen key modules strongly associated with differential immune cells, the WGCNA package (v 1.73) was used to analyze the correlation between modules and differential immune cells (|cor| > 0.3, p < 0.05). All modules meeting the criteria of |cor| > 0.3 and p < 0.05 were selected as key modules, and genes within these key modules were recorded as module genes.

Finally, the functions geneModuleMembership and geneTraitSignificance were used to analyze genes within the key modules. Genes subjected to both module membership (MM) analysis and gene significance (GS) analysis were identified as key module genes, i.e., the obtained ICRGs.

### 2.4 Identification of candidate genes

The intersection of DEGs, TRGs and ICRGs (candidate genes) was analyzed using the ggvenn package (v 0.1.10) (Zheng et al., 2022). GO enrichment analysis is a method for functional annotation of genes using the GO database, which includes three parts: BP, CC, and MF. The KEGG pathway enrichment analysis uses the KEGG database to perform pathway annotation on all identified proteins (including screened differentially expressed proteins), and analyze the main metabolic pathways and signal transduction pathways involved in these proteins or genes. GO and KEGG enrichment analyses were performed on candidate genes using the clusterProfiler package (v 4.2.2) (p < 0.05) (Wu et al., 2021). Candidate genes were imported into the STRING database to construct a PPI network (interaction score >0.15). The network was visualized using Cytoscape software (v 3.10.3) (Shannon et al., 2003).

### 2.5 Identification of key genes

Machine learning is a powerful data analysis technique that has been widely used in genetic feature screening. To screen the obtained core genes, two algorithms were used: the LASSO regression and SVM-RFE. LASSO regression was analyzed using the glmnet package (v 4.1.8) (Friedman et al., 2010), and 5-fold cross-validation was performed to determine the best lambda value to screen out core genes in group 1 at the minimum error. Then, based on the candidate genes, the SVM-RFE model was established using the caret package (v 6.0-94) (López-Díaz et al., 2022), and the non-significant features were gradually eliminated through 5-fold cross-validation. The model performance of each step was evaluated to select the model with the highest possible accuracy and lowest possible error rate to obtain core genes in group 2. Finally, the ggvenn package (v 0.1.10) was used to take the intersection of core genes in group 1 and core genes in group 2, which was defined as the core genes.

To further confirm the expression levels of the core genes, a Wilcoxon test (p < 0.05) was used. The expression of core genes was validated in all samples from the GSE12852 and the GSE53868 datasets. Boxplots were drawn using the ggplot2 package (v 3.5.1) to show the results. Genes that showed significant differences and consistent expression trends in both datasets were selected as candidate key genes in this study for subsequent analysis.

To evaluate the ability of candidate key genes to distinguish between POP and control tissue samples, ROC analysis was performed using the pROC package (v 1.18.5) (Robin et al., 2011)based on candidate key genes in the GSE12852 and GSE53868 datasets, with the calculation of AUC values. An AUC value exceeding 0.7 was considered indicative of good sample discrimination ability of the gene. Finally, genes with an AUC value greater than 0.7 in both datasets were selected as key genes.

### 2.6 Construction of nomogram model

To evaluate the ability of key genes to predict POP, a nomogram of key genes was created using the rms package (v 6.8.1) (Yan et al., 2024) based on all samples in the GSE12852 dataset to predict the incidence of POP. The diagnostic performance of the nomogram was evaluated by calculating the AUC of the ROC curve. An AUC >0.7, with higher AUC value indicated better predictive performance of the nomogram model. Among them, the ROC curve was plotted using the pROC package (v 1.18.5).

### 2.7 Correlation analysis

To assess the correlations among the key genes, a Spearman correlation analysis (|correlation coefficient (r)| > 0.3, p < 0.05) was performed using the psych package (v 2.4.6.26) across all samples in the GSE12852 dataset. Additionally, visualization was performed using the ggplot2 package (v 3.5.1) (Robles-Jimenez et al., 2021).

### 2.8 Gene set enrichment analysis (GSEA)

To further understand the biological functions and pathways related to POP of key genes in all samples of the GSE12852 dataset, the gene set c2. cp.kegg.v7.4. symbols.gmt downloaded from the MsigDB, was used as the reference gene set. The cor function in stats package (v 4.3.3) was employed to perform Spearman correlation analysis between each key gene and all other genes, respectively, to obtain the correlation coefficients of the genes. These coefficients were then sorted from big to small, and GSEA was conducted using the clusterProfiler package (v 4.2.2). The thresholds were set as |NES| > 1 and p < 0.05. Finally, the enrichplot package (v 1.20.3) was utilized to visualize the GSEA results (Li H. et al., 2022).

### 2.9 Molecular regulatory network analysis

To investigate the potential regulatory mechanisms of key genes, the ENCORI was used to predict potential candidate miRNAs of the key genes. The ChEA3 database was employed to predict candidate TFs of the biomarkers. Cytoscape software (v 3.10.3) was utilized to construct the miRNA-mRNA interaction network diagram and TF-mRNA interaction network diagram, respectively.

### 2.10 Drug prediction

To explore potential drugs for POP, predictions of drugs or compounds were performed using the DsigDB based on the obtained key genes. Additionally, the Cytoscape software (v 3.10.2) was used to visualize the drug or compound-biomarker interaction network.

### 2.11 Reverse transcription-quantitative polymerase chain reaction (RT-qPCR) validation of key genes

To verify whether the expression of key genes in clinical samples was consistent with the bioinformatics results, tissue samples were collected from 5 controls and 5 POP patients who were recruited from Gansu Provincial Hospital. All participants signed and filled in the informed consent form, and the ethical approval agency was Ethics committee of Gansu Provincial Hospital and No. 2025-676. Graphpad Prism 10 was used to plot the results and calculate p values. Total RNA was extracted from 10 samples using the Trizol method (Vazyme Biotechnology Co., Nanjing, China). Total RNA was reverse transcribed into cDNA using the HP All-in-one qRT Master Mix II RT203-Ver.1 kit (Yungeng Biotechnology Co., Kunming, China). Subsequent RT-qPCR was performed using 2 x Universal Blue SYBR Green qPCR Master Mix (Servicebio, Wuhan, China) with GAPDH as an internal reference to quantify the expression of prognostic genes. The PCR primer sequences are shown in Supplementary Table 2. The expression levels of prognostic genes in the 2 groups were calculated using the 2^−ΔΔCt^ algorithm, and the expression levels were compared using the t-test (p < 0.05).

### 2.12 Statistical analysis

The bioinformatics data were analyzed using R software (v 4.3.3). The Wilcoxon test was employed to assess the differences between the 2 sample groups. A p < 0.05 was considered statistically significant.

## 3 Results

### 3.1 Identification of DEGs

**FIGURE 1:**
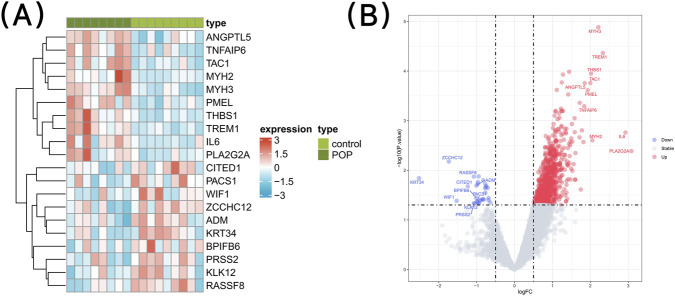
Panel A presents a nomogram withpoint scales for CCNL1,NAMPT,total points, linear predictor,and predicted value for risk assessment. Panel B hows a receiver operating characteristic (ROC) curve with an area under the curve (AUC) of zero point eight four seven, illustrating sensitivity versus specificity.

Differential expression analysis showed that there were 864 DEGs between the POP and the control samples in the GSE12852 dataset (p < 0.05 and |log_2_FC| > 0.5). Among them, 833 genes were upregulated and 31 genes were downregulated in the POP compared with the control samples (Figures 1A,B) (Supplementary Table 3).

### 3.2 Acquisition of 420 ICRGs

**FIGURE 2:**
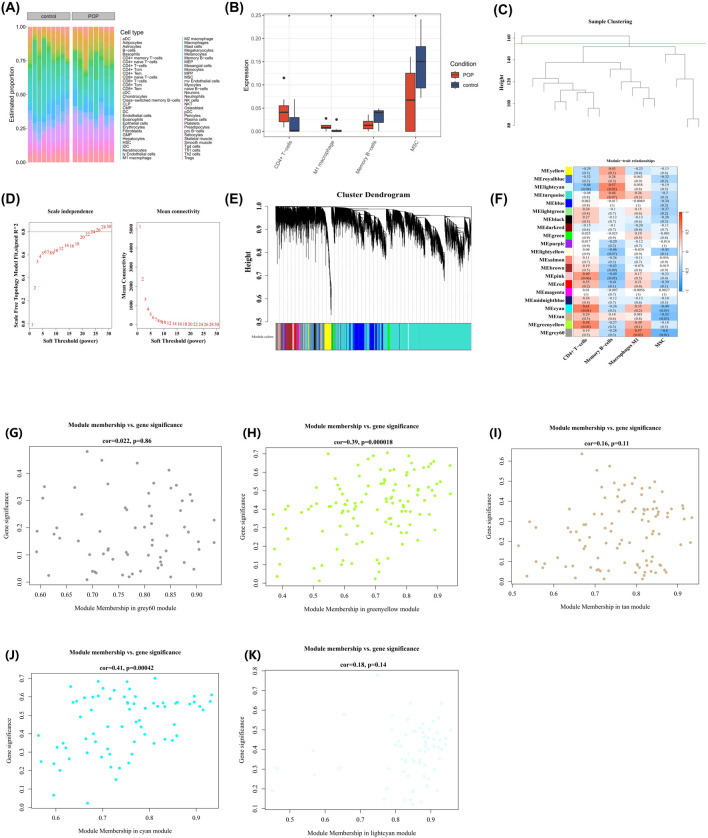
Immune cell infiltration profiling and WGCNA-based identification of immune checkpoint-related genes (ICRGs); **(A)** Infiltration abundance of immune cells; **(B)** Identification of differentially infiltrated immune cells; **(C)** Sample hierarchical clustering; **(D)** Optimization of scale-free topology parameters; **(E)** Hierarchical clustering dendrogram of co-expression modules; **(F)** Module-immune cell correlation heatmap; **(G)** Module membership-gene significance (MM-GS) analysis of MEgrey60; **(H)** MM-GS correlation of MEgreenyellow module; **(I)** MM-GS distribution in MEtan module; **(J)** MM-GS relationship in MEcyan module; **(K)** MM-GS visualization for MElightcyan module.

To obtain ICRGs, the infiltration abundances of 64 types of immune cells between the POP and control samples were evaluated in the GSE12852 dataset (Figure 2A). The Wilcoxon test (p < 0.05) was then applied, and results showed significant infiltration differences in 4 types of immune cells. Among them, the immune cells significantly upregulated in POP samples were: CD4 + T cells and M1 macrophages; the immune cells significantly downregulated in POP samples were: memory B cells and mesenchymal stem cells (MSCs) (Figure 2B). These 4 immune cells with differential infiltration were selected for subsequent analysis.

Then, WGCNA was performed on the expression matrices of all samples in the GSE12852 dataset. First, hierarchical clustering analysis was performed, and one outlier sample was removed (classified according to the red line with a height of 155). The remaining samples were regarded as clustered samples. (Figure 2C). Secondly, the power threshold β was determined to be 24 because it was the lowest power that satisfied the scale-free topology criterion (*R* ^2^ ≈ 0.80), ensuring that the constructed network accurately reflected the biological scale-free property of gene co-expression. At this time, the ordinate *R* ^2^ was around 0.80, indicating that the network was getting closer to a scale-free distribution, and the mean value of the adjacency function was gradually approaching 0, showing a gentle trend (Figure 2D). Thirdly, a total of 21 modules (excluding the gray module) were obtained through TOM-based hierarchical clustering (Figure 2E). Finally, in the correlation analysis between modules and differentially expressed immune cells, we selected all modules meeting the criteria of |cor| > 0.3 and p < 0.05, which were designated as key modules. Among them, MEgrey60 showed the strongest correlations with M1 macrophages (cor = 0.57, p = 0.02) and MSCs (cor = −0.6, p = 0.01); MEgreenyellow had the strongest correlation with CD4 + T cells (cor = 0.58, p = 0.02); MEtan showed the strongest correlation with MSCs (cor = −0.53, p = 0.03); MEcyan had the strongest correlation with CD4 + T cells (cor = 0.61, p = 0.01); and MElightcyan showed the strongest correlation with memory B cells (cor = 0.57, p = 0.02) (Figure 2F). In addition, MM and GS analyses were performed on the genes included in the key modules. The results showed that MEgrey60 contained 65 genes (Figure 2G) (Supplementary Table 4); MEgreenyellow contained 114 genes (Figure 2H) (Supplementary Table 5); MEtan contained 103 genes (Figure 2I) (Supplementary Table 6); MEcyan contained 70 genes (Figure 2J) (Supplementary Table 7); and MElightcyan contained 68 genes (Figure 2K) (Supplementary Table 8). All key modules collectively included 420 genes, which were recorded as the final ICRGs.

### 3.3 Acquisition of six candidate genes

**FIGURE 3:**
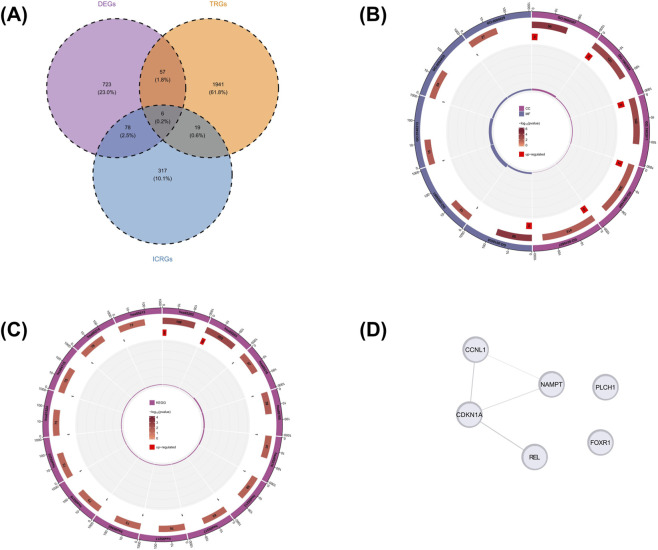
Identification and functional analysis of candidate genes at the intersection of DEGs, TRGs, and ICRGs. **(A)** Venn diagram showing the overlap of DEGs, TRGs, and ICRGs ; **(B)** GO enrichment analysis of the six candidate genes ; **(C)** KEGG pathway enrichment analysis of the six candidate genes; **(D)** PPI network of the six candidate genes.

Next, the intersection of 864 DEGs, 2023 TRGs and 420 ICRGs was taken, and a total of six candidate genes were obtained for subsequent analysis (Figure 3A). GO enrichment analyses showed that six candidate genes were enriched in 17 GO signaling pathways (p < 0.05). Among them, there were 5 CC pathways, including cyclin-dependent protein kinase holoenzyme complex, serine/threonine protein kinas complex, etc., there were 12 MF pathways, including cyclin-dependent protein serine/threonine kinase regulator activity, molecular sequestering activity. Notably, no terms reached significance for Biological Process (BP) under the current threshold (Figure 3B) (Supplementary Table 9). These genes were enriched in 22 KEGG signaling pathways (p < 0.05), including transcriptional misregulation in cancer, viral carcinogenesis, etc. (Figure 3C) (Supplementary Table 10).

Subsequently, a PPI network containing six key nodes and 4 interactions was constructed, suggesting that there might be relatively strong interaction relationships among these six proteins. These interactions included the interaction between CCNL1 and NAMPT, as well as between CDKN1A and REL, etc. (Figure 3D).

### 3.4 Identification of 2 key genes

**FIGURE 4:**
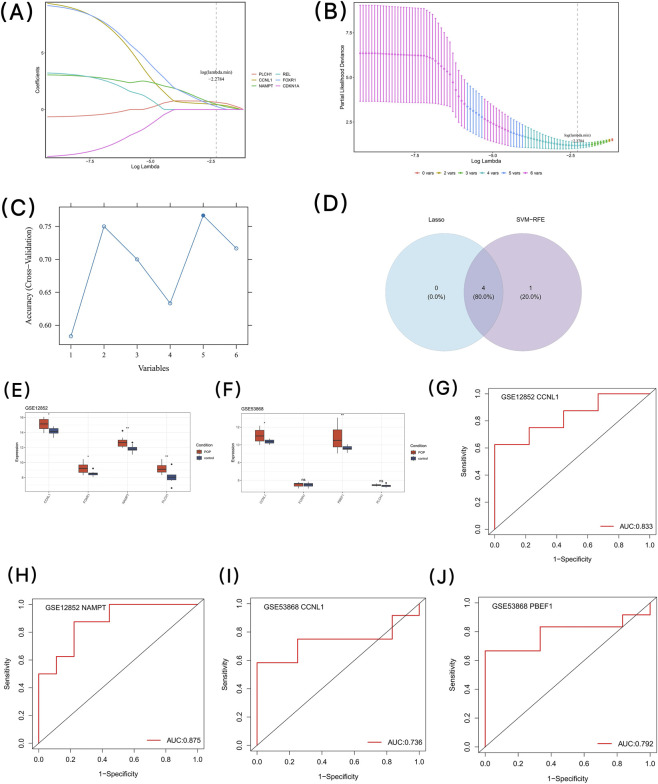
Machine learning-based identification and validation of key genes associated with POP; **(A)** LASSO coefficient profiles of candidate genes; **(B)** Cross-validation for tuning parameter selection in the LASSO model; **(C)** Feature selection using the SVM-RFE algorithm. Figure; **(D)** Venn diagram of core genes identified by LASSO and SVM-RFE; **(E)** Validation of core gene expression in the GSE12852 dataset; **(F)** Validation of core gene expression in the GSE53868 dataset; **(G)** ROC curve for CCNL1 in the GSE12852 dataset; **(H)** ROC curve for NAMPT in the GSE12852 dataset; **(I)** ROC curve for CCNL1 in the GSE53868 dataset; **(J)** ROC curve for NAMPT in the GSE53868 dataset.

Using the Lasso algorithm, the optimal log value of lambda (log(lambda.min)) was −2.2784, and a total of 4 core genes in group 1 were screened, namely, PLCH1, CCNL1, NAMPT and FOXR1 (Figures 4A,B). A total of 5 core genes in group 2 (PLCH1, CCNL1, NAMPT, FOXR1 and REL) were screened by establishing the SVM-RFE model (Figure 4C). Subsequently, the intersection of the 4 core genes in group 1 and the 5 core genes in group 2 was obtained, resulting in a total of 4 core genes (Figure 4D).

Using the Wilcoxon test, the expression of the core genes was verified in all samples of the GSE12852 and the GSE53868 datasets. In the GSE12852 dataset, the expression levels of the 4 core genes, namely, PLCH1, CCNL1, NAMPT and FOXR1, were significantly upregulated in the POP samples (p < 0.05) (Figure 4E). In the GSE53868 dataset, only the core genes CCNL1 and NAMPT (also known as PBEF1) showed significant expression levels (p < 0.05) (Figure 4F), and their expression trends were consistent with those in the GSE12852 dataset. Therefore, CCNL1 and NAMPT were determined as the candidate key genes.

ROC analysis showed that in the GSE12852 dataset, the AUC value of CCNL1 was 0.833, and the AUC value of NAMPT was 0.875 (Figures 4G,H). Similarly, in the GSE53868 dataset, the AUC values of both candidate key genes were also greater than 0.7 (Figures 4I,J). Therefore, CCNL1 and NAMPT were determined as the key genes.

### 3.5 Construct a nomogram model of 2 key genes

**FIGURE 5:**
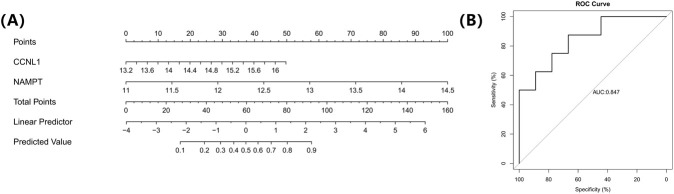
Development and validation of a nomogram model for POP prediction based on key genes; **(A)** Nomogram for predicting the risk of POP; **(B)** ROC curve evaluating the predictive accuracy of the nomogram.

To evaluate the clinical diagnostic value of key genes, nomograms of CCNL1 and NAMPT were created to predict the incidence of POP based on all samples in the GSE12852 dataset (Figure 5A). The AUC of the ROC curve was 0.847 (Figure 5B), which further indicated the high prediction accuracy of the nomogram.

### 3.6 Correlation and biological pathways of 2 key genes

**FIGURE 6:**
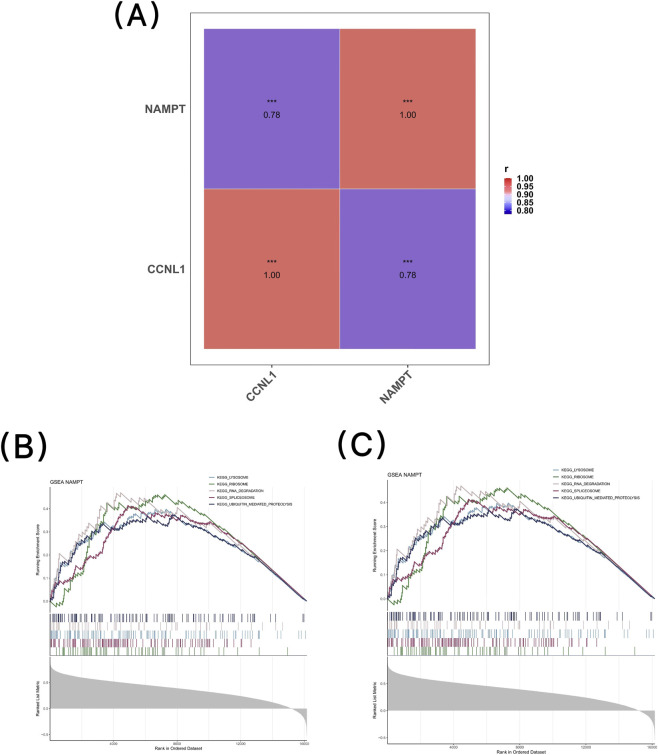
Correlation and functional enrichment of the key genes CCNL1 and NAMPT; **(A)** Correlation analysis between CCNL1 and NAMPT expression. **(B)** Gene Set Enrichment Analysis (GSEA) for CCNL1; **(C)** Gene Set Enrichment Analysis (GSEA) for NAMPT.

Correlation analysis showed that there was a strong correlation between CCNL1 and NAMPT (r = 0.78, p < 0.001) (Figure 6A).

GSEA showed that CCNL1 was enriched in a total of 7 pathways, such as epithelial cell signaling in *helicobacter pylori* infection, acute myeloid leukemia, and ubiquitin mediated proteolysis (Figure 6B) (Supplementary Table 11), and NAMPT was enriched in 9 pathways, such as ribosome, spliceosome, and lysosome (|NES| > 1, p < 0.05) (Figure 6C) (Supplementary Table 12). Moreover, they jointly participated in the ubiquitin mediated proteolysis pathway. These results suggest a potential association between these pathways and the occurrence and development of POP.

### 3.7 Regulatory network of 2 key genes

**FIGURE 7:**
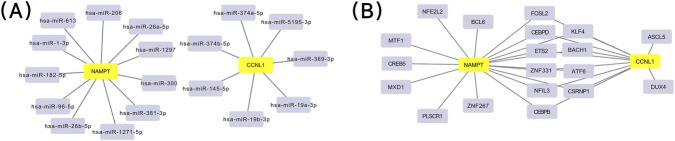
Regulatory networks of the key genes CCNL1 and NAMPT; **(A)** miRNA-mRNA interaction network. **(B)** Transcription Factor (TF)-mRNA interaction network.

First, a miRNA-mRNA network containing 20 key nodes and 18 interactions was constructed. Among them, NAMPT targeted 11 miRNAs, such as hsa-miR-206 and hsa-miR-381-3p. CCNL1 targeted 7 miRNAs, such as hsa-miR-5195-3p and hsa-miR-369-3p (Figure 7A). In addition, a TF-mRNA network with 21 key nodes and 28 interactions was built. Among these, there were 17 TFs that targeted NAMPT, including BCL6 and ZNF267, and 12 TFs that targeted CCNL1, such as ASCL5 and DUX4. Notably, 10 TFs were found to jointly target these 2 key genes, such as FOSL2 and ZNF267 (Figure 7B), which had a regulatory effect on NAMPT and CCNL1 and might influence the development of POP.

### 3.8 Drug prediction of 2 key genes

**FIGURE 8:**
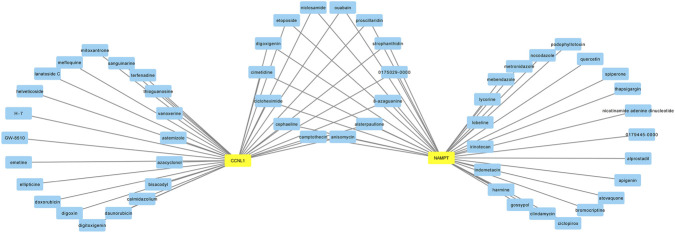
Prediction of potential targeting drugs or compounds; Drug-biomarker interaction network.

A drug or compound-biomarker interaction network containing 57 key nodes and 69 interactions was constructed. A total of 34 drugs or compounds were predicted for CCNL1, and 35 drugs or compounds were predicted for NAMPT. Among them, 14 drugs or compounds were associated with both key genes, such as niclosamide, camptothecin, and anisomycin (Figure 8). These predictions represent potential starting points for future experimental validation. These results suggested that CCNL1 and NAMPT might serve as potential targets through which these drugs or compounds influence POP.

### 3.9 Expression of 2 key genes in POP samples

**FIGURE 9:**
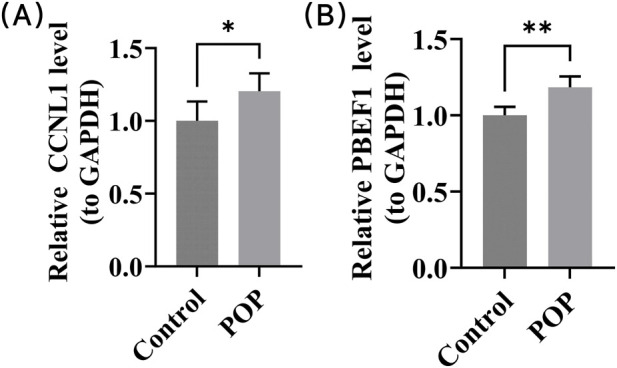
RT-qPCR analysis confirms high expression of key genes in POP clinical samples; **(A)** Elevated CCNL1 expression levels in POP tissues; **(B)** Elevated NAMPT expression levels in POP tissues.

Clinical samples were used for RT-qPCR validation of CCNL1 and NAMPT. The results confirmed that CCNL1 and NAMPT were expressed at significantly high levels in POP samples (p < 0.05) (Figures 9A,B). In summary, the RT-qPCR validation of the key genes was consistent with the bioinformatics analysis and thus provided valuable insights into the gene expression patterns relevant to POP patients. This might help in the development of personalized treatment strategies.

## 4 Discussion

POP is a common disease caused by the weakening of the pelvic floor support structure. Based on the known association between telomere shortening and immune cell senescence (Raju and Linder, 2021; Shi et al., 2024; Katerina, 2024), this study hypothesized that there are key molecular nodes related to both processes in POP. Through integrated bioinformatics analysis,we successfully identified CCNL1 and NAMPT as key genes associated with both immune cells and telomeres in POP. This discovery provides a new entry point for in-depth exploration of the molecular mechanism of POP from the cross - perspective of immunosenescence and replicative senescence,and lays a genetic foundation for the subsequent development of targeted intervention strategies.

CCNL1 (cyclin L1), also known as Ania - 6a, is one of the members of the cyclin family (O'Brien et al., 2022). The gene encoding CCNL1 is localized to chromosome 3q25 and produces a key regulatory factor critical for pre-mRNA processing, in which it is thought to play a central role. By modulating the G1/S phase transition, CCNL1 contributes to the regulation of cell cycle progression. It is also implicated in maintaining normal cellular physiological functions and has been associated with tumorigenesis and cancer development (Zeng et al., 2022). Studies have shown that in polycystic ovary syndrome (PCOS), overexpression of CCNL upregulates FOXO1,leading to enhanced apoptosis, diminished glucose uptake, and compromised mitochondrial function (Huang et al., 2021); It has been proposed that CCNL may modulate apoptotic processes through the regulation of its own expression, thereby contributing to the pathogenesis of pelvic organ prolapse (POP). Furthermore, studies indicate that under conditions of liver ischemia-reperfusion, genes including DNAJB2, BTG1, and CCNL1 are predominantly expressed in immune cells such as macrophages and T cells, and are strongly associated with inflammatory signaling pathways, including those mediated by TNF-α (Lai et al., 2024). Moreover, CCNL1, an inflammation-related gene, has been identified as a potential biomarker for osteoarthritis (OA) (Li S. et al., 2023)。Further research on the pelvic floor inflammatory environment has revealed an enhanced inflammatory response in the vaginal tissues of patients with pelvic organ prolapse POP. These findings suggest that significant alterations in the local inflammatory milieu may represent a key component of POP pathogenesis. In particular, the anterior vaginal wall tissue in POP patients exhibits elevated levels of various inflammatory cytokines, such as IL-1, TNF, and IFN, which may disrupt collagen metabolism and thereby contribute to the development of POP (Chen et al., 2024). In conclusion, the bioinformatics analysis suggests that CCNL1 may potentially be involved in the pathological progression of POP, possibly through modulating the expression or secretion of inflammatory factors such as IL-1 and TNF-α, which could exacerbate vaginal tissue injury and impede repair mechanisms; however, this requires experimental validation. Furthermore, the results of this study align with the RT-qPCR data, providing additional support for the proposed mechanism.

NAMPT (Nicotinamide Phosphoribosyltransferase), also known as pre-B-cell colony-enhancing factor 1 (PBEF1) or visfatin (Chu et al., 2016), serves as the rate-limiting enzyme in NAD^+^ biosynthesis from nicotinamide and participates in diverse biological processes including immune regulation, cell survival, and disease pathogenesis (Gasparrini and Audrito, 2022). It modulates the activity of multiple NAD^+^-dependent enzymes, such as sirtuins, PARPs, and CD38, thereby influencing cellular metabolism, mitochondrial function, and responses to inflammation and oxidative stress. Recognized as a biomarker of chronic inflammation (Peng et al., 2024; Martínez-Morcillo et al., 2021), NAMPT is upregulated under pathological conditions including hepatic ischemia-reperfusion injury (IRI) (Lu et al., 2023),and necrotizing enterocolitis (NEC) (Liu et al., 2023). Where its inhibition with FK866 attenuates tissue damage and inflammatory responses.

In the context of POP, NAMPT is speculated to influence disease progression, possibly by promoting a pro-inflammatory microenvironment characterized by macrophage activation and secretion of cytokines (e.g., TNF-α, IL-6, and IL-1β), which exacerbates tissue injury and fibrosis. Furthermore, NAMPT appears to regulate extracellular matrix (ECM) composition, and its inhibition downregulates ECM-associated proteins (Xu et al., 2021); Given that POP involves disruption of pelvic support structures due to ECM dysfunction (Kopij et al., 2024), NAMPT may play a key role in modulating ECM remodeling in this condition.

Additionally, NAMPT/Visfatin significantly affects gene expression patterns in reproductive cells, influencing processes critical to implantation and pregnancy maintenance (Kopij et al., 2024) and localizes to lysosomal membranes where it contributes to lysosomal function (Miki et al., 2024). These findings underscore the multifunctional nature of NAMPT and suggest its involvement in both systemic inflammatory and tissue-specific mechanistic pathways underlying POP. The reliability of these findings is supported by validation experiments such as RT-qPCR.

GSEA revealed that CCNL1 and NAMPT were co-enriched in pathways such as ubiquitin - mediated proteolysis, suggesting that these pathways may underlie the role of key genes in POP. First, the enrichment of the *Helicobacter pylori* - infected epithelial cell signaling pathway indicates that systemic pathological processes such as chronic inflammation and abnormal cell proliferation may indirectly affect the collagen homeostasis of pelvic floor tissues (Ali et al., 2025; Zhang et al., 2022). Secondly, the ubiquitin - proteasome system, as a key protein degradation pathway, has its abnormal function closely related to inflammation and ECM remodeling (Tsukamoto and Yokosawa, 2006). In the context of POP, the abnormal activation of this system may be involved in regulating the turnover of structural proteins such as collagen in pelvic floor support tissues, which is consistent with the increased proteolytic activity observed in complications after polypropylene mesh implantation (Knight et al., 2022). In addition, CCNL1 and NAMPT were also enriched in the ribosome and spliceosome pathways, respectively. The mitochondrial ribosomal genes were upregulated in POP connective tissue (Tseng et al., 2010). It is suggested that mitochondrial functional defects may participate in long - term tissue remodeling by affecting cellular energy supply. As a key complex for mRNA processing, the spliceosome may indirectly regulate protein synthesis and tissue homeostasis by influencing the alternative splicing of mRNAs of ECM components (such as collagen) (Martínez-Lumbreras et al., 2024; Gong and Xia, 2019). These enriched pathways together depict a complex network in which CCNL1 and NAMPT may participate in the pathogenesis of POP by influencing protein degradation, metabolism, and synthesis processes, and jointly disrupting the balance of the ECM.

Collective evidence from this and previous studies highlights the involvement of multiple miRNAs and signaling pathways in the pathophysiology of POP. Specifically, miR-26a-5p targets ABL2, MMP16, and PDE7A, and its inhibition attenuates fibrotic responses and improves tissue architecture, suggesting a protective role in POP development (Zhang et al., 2019). Concurrently, miR-145-5p regulates fibroblast proliferation, apoptosis, and migration under high-glucose conditions (Wang et al., 2024), while miR-5195-3p modulates ECM metabolism via the LOX/TGF-β1 axis (Deng et al., 2021), Dysregulation of these miRNAs may compromise pelvic tissue integrity through impaired collagen synthesis and ECM remodeling (Zhang et al., 2025). In addition, CSRNP1—upregulated in POP-derived vaginal fibroblasts—inhibits proliferation, promotes apoptosis, and enhances collagen degradation. Mechanistically, it is transcriptionally regulated by SNAI2, implicating the SNAI2/CSRNP1 axis as a key driver of POP (Zhu et al., 2024); Furthermore, co-expression network analysis identified immune-related modules highly associated with POP, with ZNF331 emerging as a hub gene (Zhao et al., 2020). Reinforcing the role of inflammatory processes in POP etiology.

From a biological perspective, the co-occurrence of CCNL1 and NAMPT highlights a potential crosstalk between replicative senescence and chronic inflammation in POP. Telomere shortening in pelvic fibroblasts may trigger cellular senescence, leading to the secretion of inflammatory factors (e.g., IL-1β, TNF-α) that are regulated by NAMPT (Nunes et al., 2024). Concurrently, CCNL1-mediated cell cycle arrest may impair the proliferative capacity of fibroblasts needed for ECM repair (Zeng et al., 2022). This synergistic loop of “senescence-inflammation-matrix degradation” likely represents a core mechanism driving the progression from asymptomatic pelvic floor weakness to clinically manifest prolapse.

To place our findings within the broader scientific landscape, we compared our results with recent transcriptomic studies in POP. Previous study identified significant immune cell infiltration and highlighted LOX, IL-6, SDC1, ICAM1, and CD38 as a hub gene in POP, our study also emphasized the critical role of immune-related pathways (Wu et al., 2023). Furthermore, our identification of NAMPT aligns with recent single-cell analyses (Chiang et al., 2024), which revealed ECM accumulation and implicated cell cycle regulators in disease progression. Unlike previous studies focusing primarily on extracellular matrix components, our integration of telomere biology with immune signatures provides a novel perspective on the interplay between replicative senescence and inflammation in POP pathogenesis. Additionally, our study identified NAMPT and CCNL1 as complementary nodes potentially bridging metabolic dysfunction and immune response.

These findings collectively suggest that POP arises from dysregulated miRNA activity, aberrant ECM metabolism, impaired fibroblast function, and immune involvement. Therapeutic strategies targeting these key elements—such as modulating miRNA expression or inhibiting specific pathways like SNAI2/CSRNP1—may offer promising avenues for ameliorating POP progression.

This study computationally predicted potential therapeutic drugs targeting CCNL1 and NAMPT, among which 14 were co-targeting drugs, including nicotinamide, camptothecin, anisomycin, etc. It is important to note that these findings are hypothesis-generating and require rigorous experimental validation. As the substrate and product of the NAMPT enzyme, exogenous supplementation of nicotinamide may affect NAD + biosynthesis and cellular metabolic homeostasis through a feedback regulatory mechanism (Song et al., 2025),thereby indirectly regulating the inflammatory response and ECM remodeling process mediated by NAMPT. Camptothecin is a topoisomerase I inhibitor that can induce DNA damage and cell apoptosis (Zuco et al., 2010). Its association with CCNL1 suggests that targeting the cell cycle and apoptosis pathways may be one of the strategies to intervene in the abnormal cell behavior in the pathology of POP. As a protein synthesis inhibitor and an activator of the JNK/SAPK signaling pathway (Hazzalin et al., 1998), anisomycin may regulate the fibrotic response of pelvic floor tissues by affecting the translation process of inflammation-related proteins or stress signaling pathways. Of particular importance is that some of these predicted drugs have already been used in the treatment or research of other diseases (e.g., nicotinamide for skin diseases and metabolic disorders (Zhu et al., 2023), rapamycin derivatives for cancer treatment (Mathi et al., 2025)). Their safety profiles and pharmacological properties are already somewhat understood, which greatly accelerates the feasibility of repurposing these existing drugs for POP research.

Beyond theoretical mechanisms, our findings offer tangible translational prospects. First, the high AUC values (0.833-0.875) of CCNL1 and NAMPT suggest their utility as non-invasive diagnostic biomarkers; future studies could explore their detection in vaginal secretions or blood. Second, the nomogram constructed in this study provides a practical tool for clinicians to assess POP risk based on gene expression profiles. Regarding therapeutics, while drug predictions (e.g., niclosamide, camptothecin) are currently hypothesis-generating, they highlight NAMPT and CCNL1 as druggable targets for future intervention. Targeting these nodes could potentially restore the balance between ECM synthesis and degradation, offering a disease-modifying strategy beyond mechanical support.

This study integrated transcriptomic profiling and bioinformatic analyses—including immune infiltration and WGCNA—to identify CCNL1 and NAMPT as key genes linking immune response and telomere biology in POP. Subsequent analyses, such as nomogram construction, correlation studies, GSEA, regulatory network inference, and drug prediction, provided further mechanistic and therapeutic insights into POP pathogenesis. Given the exploratory design for candidate screening, we did not apply strict multiple-testing corrections to avoid overlooking significant biological candidates. Although this may increase false-positive risk, it facilitates broader discovery. The constructed risk prediction nomogram for uterine prolapse has not undergone external validation in independent cohorts, which limits its clinical generalizability and application. Future studies should conduct external validation in large-sample, multicenter clinical cohorts to enhance the model’s reliability. Findings require confirmation via future FDR-controlled studies. Additionally, limited sample sizes and tissue heterogeneity (uterosacral ligament in GSE12852 vs. vaginal wall in GSE53868) may introduce confounding factors affecting finding generalizability. Data and methodological biases could also impact result interpretability and reliability. Although the LASSO and SVM-RFE models were optimized using cross-validation, there remains a risk of overfitting that could compromise the generalization performance of feature selection. Finally, the reliance on computational predictions (immune infiltration, miRNA/TF networks) necessitates future laboratory investigation to confirm these digital findings. Future work will validate CCNL1/NAMPT experimentally, elucidate their crosstalk, and conduct larger multi-center studies with consistent tissue sampling to confirm findings and advance POP targeted therapies.

## Data Availability

The data presented in the study are deposited in the Gene Expression Omnibus (GEO) repository, accession numbers GSE12852 and GSE53868.

## References

[B1] Ali F. Ahmad S. Abbas Q. Qasim W. Saleem M. Z. Mohsin M. (2025). Identification of potential therapeutic targets and epithelial cell signaling pathway for *Helicobacter pylori* infection using biosurfactants as a novel green antimicrobials: a network pharmacology approach. Microb. Pathog. 206, 107767. 10.1016/j.micpath.2025.107767 40480449

[B2] Aran D. Hu Z. Butte A. J. (2017). xCell: digitally portraying the tissue cellular heterogeneity landscape. Genome Biol. 18, 220. 10.1186/s13059-017-1349-1 29141660 PMC5688663

[B3] Brito L. G. O. Pereira G. M. V. Moalli P. Shynlova O. Manonai J. Weintraub A. Y. (2022). Age And/Or postmenopausal status as risk factors for pelvic organ prolapse development: systematic review with meta-analysis. Int. Urogynecol J. 33, 15–29. 10.1007/s00192-021-04953-1 34351465

[B4] Bugge C. Adams E. J. Gopinath D. Stewart F. Dembinsky M. Sobiesuo P. (2020). Pessaries (mechanical devices) for managing pelvic organ prolapse in women. Cochrane Database Syst. Rev. 11, Cd004010. 10.1002/14651858.CD004010.pub4 33207004 PMC8094172

[B5] Chen Y. Ullah A. Chen W. Xuan J. Huang X. Liang S. (2024). Cytokine modulation in pelvic organ prolapse and urinary incontinence: from molecular insights to therapeutic targets. Mol. Med. 30, 214. 10.1186/s10020-024-00989-3 39538179 PMC11562709

[B6] Chiang Y. F. Huang K. C. Huang T. C. Chen H. Y. Ali M. Al-Hendy A. (2024). Regulatory roles of NAMPT and NAD(+) metabolism in uterine leiomyoma progression: implications for ECM accumulation, stemness, and microenvironment. Redox Biol. 78, 103411. 10.1016/j.redox.2024.103411 39486360 PMC11564007

[B7] Chu M. Rong J. Wang Y. Zhu L. Xing B. Tao Y. (2016). Strong association of the polymorphisms in PBEF1 and knee OA risk: a two-stage population-based study in China. Sci. Rep. 6, 19094. 10.1038/srep19094 26752339 PMC4707545

[B8] Collins S. Lewicky-Gaupp C. (2022). Pelvic organ prolapse. Gastroenterology Clin. N. Am. 51, 177–193. 10.1016/j.gtc.2021.10.011 35135661

[B9] Deng Z. M. Dai F. F. Yuan M. Q. Yang D. Y. Zheng Y. J. Cheng Y. X. (2021). Advances in molecular mechanisms of pelvic organ prolapse. Exp. Ther. Med. 22, 1009. 10.3892/etm.2021.10442 34345291 PMC8311251

[B10] Friedman J. Hastie T. Tibshirani R. (2010). Regularization paths for generalized linear models via coordinate descent. J. Stat. Softw. 33, 1–22. 10.18637/jss.v033.i01 20808728 PMC2929880

[B11] Gasparrini M. Audrito V. (2022). NAMPT: a critical driver and therapeutic target for cancer. Int. J. Biochem. Cell. Biol. 145, 106189. 10.1016/j.biocel.2022.106189 35219878

[B12] Gong R. Xia Z. (2019). Collagen changes in pelvic support tissues in women with pelvic organ prolapse. Eur. J. Obstet. Gynecol. Reprod. Biol. 234, 185–189. 10.1016/j.ejogrb.2019.01.012 30710765

[B13] Hazzalin C. A. Le Panse R. Cano E. Mahadevan L. C. (1998). Anisomycin selectively desensitizes signalling components involved in stress kinase activation and fos and Jun induction. Mol. Cell. Biol. 18, 1844–1854. 10.1128/mcb.18.4.1844 9528756 PMC121414

[B14] Huang J. Zhao J. Geng X. Chu W. Li S. Chen Z. J. (2021). Long non-coding RNA lnc-CCNL1-3:1 promotes granulosa cell apoptosis and suppresses glucose uptake in women with polycystic ovary syndrome. Mol. Ther. Nucleic Acids 23, 614–628. 10.1016/j.omtn.2020.12.008 33552682 PMC7819816

[B15] Katerina S. (2024). Telomeres and immunodeficiencies. Hum. Immunol. 85, 111146. 10.1016/j.humimm.2024.111146 39317127

[B16] Kim J. J. Ahn A. Ying J. Y. Pollens-Voigt J. Ludlow A. T. (2025). Effect of aging and exercise on hTERT expression in thymus tissue of hTERT transgenic bacterial artificial chromosome mice. Geroscience 47, 3325–3341. 10.1007/s11357-024-01319-5 39222198 PMC12181544

[B17] Knight K. M. King G. E. Palcsey S. L. Suda A. Liang R. Moalli P. A. (2022). Mesh deformation: a mechanism underlying polypropylene prolapse mesh complications in vivo. Acta Biomater. 148, 323–335. 10.1016/j.actbio.2022.05.051 35671876 PMC9453339

[B18] Kopij G. Kiezun M. Dobrzyn K. Zaobidna E. Zarzecka B. Rak A. (2024). Visfatin affects the transcriptome of porcine luteal cells during early pregnancy. Int. J. Mol. Sci. 25, 2339. 10.3390/ijms25042339 38397019 PMC10889815

[B19] Lai W. Yu J. Wen D. (2024). Diagnosis and molecular characterization of potential RNA binding protein involved in the pathogenesis of liver ischemia reperfusion injury. J. Inflamm. Res. 17, 4881–4893. 10.2147/JIR.S468828 39070133 PMC11278829

[B20] Langfelder P. Horvath S. (2008). WGCNA: an R package for weighted correlation network analysis. BMC Bioinforma. 9, 559. 10.1186/1471-2105-9-559 19114008 PMC2631488

[B21] Li S. C. Jia Z. K. Yang J. J. Ning X. H. (2022a). Telomere-related gene risk model for prognosis and drug treatment efficiency prediction in kidney cancer. Front. Immunol. 13, 975057. 10.3389/fimmu.2022.975057 36189312 PMC9523360

[B22] Li H. Gao L. Du J. Ma T. Ye Z. Li Z. (2022b). Differentially expressed gene profiles and associated ceRNA network in ATG7-Deficient lens epithelial cells under oxidative stress. Front. Genet. 13, 1088943. 10.3389/fgene.2022.1088943 36568386 PMC9768497

[B23] Li Y. Kong M. Wang J. Han P. Zhang N. Yang X. (2023a). Exercise-induced circulating exosomes potentially prevent pelvic organ prolapse in clinical practice via inhibition of smooth muscle apoptosis. Heliyon 9, e12583. 10.1016/j.heliyon.2022.e12583 37077375 PMC10106923

[B24] Li S. Ma L. Cui R. (2023b). Identification of novel diagnostic biomarkers and classification patterns for osteoarthritis by analyzing a specific set of genes related to inflammation. Inflammation 46, 2193–2208. 10.1007/s10753-023-01871-w 37462886

[B25] Liang T. Chen J. Xu G. Zhang Z. Xue J. Zeng H. (2022). STAT1 and CXCL10 involve in M1 macrophage polarization that May affect osteolysis and bone remodeling in extrapulmonary tuberculosis. Gene 809, 146040. 10.1016/j.gene.2021.146040 34710525

[B26] Ling L. Li B. Wu H. Zhang K. Li S. Ke B. (2024). Construction and validation of molecular subtype and signature of immune cell-related telomeric genes and prediction of prognosis and immunotherapy efficacy in ovarian cancer patients. J. Gene Med. 26, e3606. 10.1002/jgm.3606 38282157

[B27] Liu Q. Gao K. Ding X. Mo D. Guo H. Chen B. (2023). NAMPT inhibition relieves intestinal inflammation by regulating macrophage activation in experimental necrotizing enterocolitis. Biomed. Pharmacother. 165, 115012. 10.1016/j.biopha.2023.115012 37329710

[B28] Liu X. Su M. Wei L. Zhang J. Wang W. Hao Q. (2024). Single-cell analysis of uterosacral ligament revealed cellular heterogeneity in women with pelvic organ prolapse. Commun. Biol. 7, 159. 10.1038/s42003-024-05808-3 38326542 PMC10850063

[B29] López-Díaz Jó M. Méndez-González J. López-Serrano P. M. Sánchez-Pérez F. d. J. Méndez-Encina F. M. Mendieta-Oviedo R. (2022). Dummy regression to predict dry fiber in Agave lechuguilla torr. In two large-scale bioclimatic regions in Mexico. PLoS One 17, e0274641 10.1371/journal.pone.0274641 36108072 PMC9477326

[B30] Lu J. Wang M. Chen Y. Song H. Wen D. Tu J. (2023). NAMPT inhibition reduces macrophage inflammation through the NAD+/PARP1 pathway to attenuate liver ischemia-reperfusion injury. Chem. Biol. Interact. 369, 110294. 10.1016/j.cbi.2022.110294 36460127

[B31] Martínez-Lumbreras S. Morguet C. Sattler M. (2024). Dynamic interactions drive early spliceosome assembly. Curr. Opin. Struct. Biol. 88, 102907. 10.1016/j.sbi.2024.102907 39168044

[B32] Martínez-Morcillo F. J. Cantón-Sandoval J. Martínez-Menchón T. Corbalán-Vélez R. Mesa-Del-Castillo P. Pérez-Oliva A. B. (2021). Non-canonical roles of NAMPT and PARP in inflammation. Dev. Comp. Immunol. 115, 103881. 10.1016/j.dci.2020.103881 33038343

[B33] Mathi G. R. Lee B. S. Chun Y. Shin S. Kweon S. Go A. (2025). Design, synthesis and biological evaluation of camptothecin analogue FL118 as a payload for antibody-drug conjugates in targeted cancer therapy. Bioorg Med. Chem. Lett. 118, 130085. 10.1016/j.bmcl.2024.130085 39732148

[B34] Miao Y. Wen J. Wang L. Wen Q. Cheng J. Zhao Z. (2023). scRNA-seq reveals aging-related immune cell types and regulators in vaginal wall from elderly women with pelvic organ prolapse. Front. Immunol. 14, 1084516. 10.3389/fimmu.2023.1084516 36891295 PMC9986331

[B35] Miki K. Yagi M. Kang D. Kunisaki Y. Yoshimoto K. Uchiumi T. (2024). Glucose starvation causes ferroptosis-mediated lysosomal dysfunction. iScience 27, 109735. 10.1016/j.isci.2024.109735 38706843 PMC11067335

[B36] Nunes P. R. Pereira D. A. Passeti L. F. P. Coura L. L. F. Gomes K. B. Sandrim V. C. (2024). The interplay between extracellular NAMPT and inflammatory cytokines in preeclampsia. J. Reprod. Immunol. 163, 104248. 10.1016/j.jri.2024.104248 38703439

[B37] O'Brien S. Kelso S. Steinhart Z. Orlicky S. Mis M. Kim Y. (2022). SCF(FBXW7) regulates G2-M progression through control of CCNL1 ubiquitination. EMBO Rep. 23, e55044. 10.15252/embr.202255044 36278408 PMC9724663

[B38] Peng A. Li J. Xing J. Yao Y. Niu X. Zhang K. (2024). The function of nicotinamide phosphoribosyl transferase (NAMPT) and its role in diseases. Front. Mol. Biosci. 11, 1480617. 10.3389/fmolb.2024.1480617 39513038 PMC11540786

[B39] Raju R. Linder B. J. (2021). Evaluation and management of pelvic organ prolapse. Mayo Clin. Proc. 96, 3122–3129. 10.1016/j.mayocp.2021.09.005 34863399

[B40] Ritchie M. E. Phipson B. Wu D. Hu Y. Law C. W. Shi W. (2015). Limma powers differential expression analyses for RNA-Sequencing and microarray studies. Nucleic Acids Res. 43, e47. 10.1093/nar/gkv007 25605792 PMC4402510

[B41] Robin X. Turck N. Hainard A. Tiberti N. Lisacek F. Sanchez J. C. (2011). pROC: an open-source package for R and S+ to analyze and compare ROC curves. BMC Bioinforma. 12, 77. 10.1186/1471-2105-12-77 21414208 PMC3068975

[B42] Robles-Jimenez L. E. Aranda-Aguirre E. Castelan-Ortega O. A. Shettino-Bermudez B. S. Ortiz-Salinas R. Miranda M. (2021). Worldwide traceability of antibiotic residues from livestock in wastewater and soil: a systematic review. Anim. (Basel) 12, 60. 10.3390/ani12010060 PMC8749557 35011166

[B43] Rossiello F. Jurk D. Passos J. F. d'Adda di Fagagna F. (2022). Telomere dysfunction in ageing and age-related diseases. Nat. Cell. Biol. 24, 135–147. 10.1038/s41556-022-00842-x 35165420 PMC8985209

[B44] Saraswati S. Martínez P. Serrano R. Mejías D. Graña-Castro O. Álvarez Díaz R. (2024). Renal fibroblasts are involved in fibrogenic changes in kidney fibrosis associated with dysfunctional telomeres. Exp. Mol. Med. 56, 2216–2230. 10.1038/s12276-024-01318-8 39349834 PMC11541748

[B45] Shannon P. Markiel A. Ozier O. Baliga N. S. Wang J. T. Ramage D. (2003). Cytoscape: a software environment for integrated models of biomolecular interaction networks. Genome Res. 13, 2498–2504. 10.1101/gr.1239303 14597658 PMC403769

[B46] Shi H. Z. Wang M. W. Huang Y. S. Liu Z. Li L. Wan L. P. (2024). A telomere-related gene risk model for predicting prognosis and treatment response in acute myeloid leukemia. Heliyon 10, e31705. 10.1016/j.heliyon.2024.e31705 38845982 PMC11153201

[B47] Song W. S. Shen X. Du K. Ramirez C. B. Park S. H. Cao Y. (2025). Nicotinic acid riboside maintains NAD(+) homeostasis and ameliorates aging-associated NAD(+) decline. Cell. Metab. 37, 1499–1514.e1494. 10.1016/j.cmet.2025.05.004 40315855 PMC12328062

[B48] Tseng L. H. Chen I. Lin Y. H. Chen M. Y. Lo T. S. Lee C. L. (2010). Genome-based expression profiles study for the pathogenesis of pelvic organ prolapse: an array of 33 genes model. Int. Urogynecol J. 21, 79–84. 10.1007/s00192-009-0990-y 19756343

[B49] Tsukamoto S. Yokosawa H. (2006). Natural products inhibiting the ubiquitin-proteasome proteolytic pathway, a target for drug development. Curr. Med. Chem. 13, 745–754. 10.2174/092986706776055571 16611064

[B50] Uryga A. K. Grootaert M. O. J. Garrido A. M. Oc S. Foote K. Chappell J. (2021). Telomere damage promotes vascular smooth muscle cell senescence and immune cell recruitment after vessel injury. Commun. Biol. 4, 611. 10.1038/s42003-021-02123-z 34021256 PMC8140103

[B51] van der Vaart L. R. Vollebregt A. Milani A. L. Lagro-Janssen A. L. Duijnhoven R. G. Roovers J. P. W. R. (2022). Effect of pessary vs surgery on patient-reported improvement in patients with symptomatic pelvic organ prolapse. Jama 328, 2312. 10.1001/jama.2022.22385 36538310 PMC9857016

[B52] Wang C. Huang L. Li J. Liu D. Wu B. (2024). MicroRNA miR-145-5p inhibits cutaneous wound healing by targeting PDGFD in diabetic foot ulcer. Biochem. Genet. 62, 2437–2454. 10.1007/s10528-023-10551-1 37950842

[B53] Wu T. Hu E. Xu S. Chen M. Guo P. Dai Z. (2021). clusterProfiler 4.0: a universal enrichment tool for interpreting omics data. Innov. (Camb) 2 (3), 100141. 10.1016/j.xinn.2021.100141 PMC8454663 34557778

[B54] Wu C. Zhou Z. Yang Y. Li H. Guo Y. Tong X. (2023). Bioinformatically deciphering immune cell infiltration and signature genes in pelvic organ prolapse. Int. Urogynecol J. 34, 1091–1101. 10.1007/s00192-022-05378-0 36208338

[B55] Xu Q. Li B. Wang Y. Wang C. Feng S. Xue L. (2021). Identification of VCAN as hub gene for diabetic kidney disease immune injury using integrated bioinformatics analysis. Front. Physiol. 12, 651690. 10.3389/fphys.2021.651690 34557107 PMC8454927

[B56] Yan J. Liu Y. Liu T. Zhu Q. (2024). A predictive and prognostic model for metastasis risk and prognostic factors in gastrointestinal signet ring cell carcinoma. Eur. J. Med. Res. 29, 545. 10.1186/s40001-024-02135-5 39538294 PMC11562313

[B57] Yuan S. M. Chen X. Qu Y. Q. Zhang M. Y. (2024). C6 and KLRG2 are pyroptosis subtype-related prognostic biomarkers and correlated with tumor-infiltrating lymphocytes in lung adenocarcinoma. Sci. Rep. 14, 24861. 10.1038/s41598-024-75650-4 39438534 PMC11496652

[B58] Zarzecka J. Pycek M. Pietrzykowska-Szczubelek K. Barcz E. Pomian A. (2024). Influence of pregnancy and mode of delivery on pelvic floor function: a review of literature. Ginekol. Pol. 95, 830–834. 10.5603/gpl.98418 38717218

[B59] Zeng X. Hu Z. Shen Y. Wei X. Gan J. Liu Z. (2022). MiR-5195-3p functions as a tumor suppressor in prostate cancer via targeting CCNL1. Cell. Mol. Biol. Lett. 27, 25. 10.1186/s11658-022-00326-8 35260070 PMC8905902

[B60] Zhang A. Wang H. Wang B. Yuan Y. Klein J. D. Wang X. H. (2019). Exogenous miR-26a suppresses muscle wasting and renal fibrosis in obstructive kidney disease. Faseb J. 33, 13590–13601. 10.1096/fj.201900884R 31593640 PMC6894078

[B61] Zhang J. Wang W. Yan S. Li J. Wei H. Zhao W. (2022). CagA and VacA inhibit gastric mucosal epithelial cell autophagy and promote the progression of gastric precancerous lesions. Zhong Nan Da Xue Xue Bao Yi Xue Ban. 47, 942–951. 10.11817/j.issn.1672-7347.2022.210779 36039592 PMC10930283

[B62] Zhang H. Kong W. Xie Y. Zhao X. Luo D. Chen S. (2023). Telomere-related genes as potential biomarkers to predict endometriosis and immune response: development of a machine learning-based risk model. Front. Med. (Lausanne) 10, 1132676. 10.3389/fmed.2023.1132676 36968845 PMC10034389

[B63] Zhang R. Li Y. Zhang J. (2024). Molecular mechanisms of pelvic organ prolapse influenced by FBLN5 via FOSL1/miR-222/MEIS1/COL3A1 axis. Cell. Signal 114, 111000. 10.1016/j.cellsig.2023.111000 38056607

[B64] Zhang H. Wang X. Dong M. Wang J. Ren W. (2025). Unveiling novel regulatory mechanisms of miR-5195-3p in pelvic organ prolapse pathogenesis. Biol. Reprod. 112, 86–101. 10.1093/biolre/ioae162 39530351

[B65] Zhao Y. Xia Z. Lin T. Yin Y. (2020). Significance of hub genes and immune cell infiltration identified by bioinformatics analysis in pelvic organ prolapse. PeerJ 8, e9773. 10.7717/peerj.9773 32874785 PMC7441923

[B66] Zheng Y. Gao W. Zhang Q. Cheng X. Liu Y. Qi Z. (2022). Ferroptosis and autophagy-related genes in the pathogenesis of ischemic cardiomyopathy. Front. Cardiovasc Med. 9, 906753. 10.3389/fcvm.2022.906753 35845045 PMC9279674

[B67] Zhou Q. Lu M. Li G. S. Peng G. L. Song Y. F. (2023). Identification of potential molecular mechanisms and therapeutic targets for recurrent pelvic organ prolapse. Heliyon 9, e19440. 10.1016/j.heliyon.2023.e19440 37681155 PMC10481308

[B68] Zhu J. R. Wang J. Wang S. S. (2023). A single-center, randomized, controlled study on the efficacy of niacinamide-containing body emollients combined with cleansing gel in the treatment of mild atopic dermatitis. Skin. Res. Technol. 29, e13475. 10.1111/srt.13475 37753690 PMC10509598

[B69] Zhu J. Xu H. N. Lin T. Xia Z. J. (2024). Silencing of cysteine and serine rich nuclear protein 1 inhibits apoptosis, senescence and collagen degradation in human-derived vaginal fibroblasts in response to oxidative stress or DNA damage. Exp. Cell. Res. 440, 114139. 10.1016/j.yexcr.2024.114139 38908423

[B70] Zuco V. Benedetti V. Zunino F. (2010). ATM- and ATR-Mediated response to DNA damage induced by a novel camptothecin, ST1968. Cancer Lett. 292, 186–196. 10.1016/j.canlet.2009.12.001 20042274

